# Endoscopic ultrasound-guided hepaticojejunostomy using forward-viewing echoendoscope for hepatic duct obstruction due to postoperative bile duct injury

**DOI:** 10.1055/a-2545-2689

**Published:** 2025-03-12

**Authors:** Kei Yane, Kota Hanada, Kotaro Morita, Koki Yoshida, Sota Hirokawa, Yuki Ikeda, Tetsuya Sumiyoshi

**Affiliations:** 1 36737 Department of Gastroenterology, Tonan Hospital, Sapporo, Japan

Bile duct injuries can occur at various sites, and the Strasberg classification is useful in considering their treatment strategy 1 . A Strasberg Type B injury is a bile duct obstruction that commonly occurs from the atypical right hepatic duct, and its endoscopic treatment is difficult 2 .

A 70-year-old man who underwent pancreaticoduodenectomy for ampullary carcinoma was referred to our department because of abdominal pain and fever. Computed tomography and magnetic resonance cholangiopancreatography showed a fluid collection on the liver surface and dilation of the right anterior hepatic duct, resulting in a diagnosis of cholangitis and bile leakage ( Fig. 1 ). Although a double-balloon endoscope-assisted endoscopic retrograde cholangiopancreatography (ERCP) was performed, the right anterior hepatic duct could not be identified ( Fig. 2 ). Percutaneous transhepatic biliary drainage (PTBD) was performed, resulting in symptomatic improvement ( Fig. 3 ). A review of the preoperative images revealed a variation in the right anterior bile duct, which was ligated during surgery, resulting in complete occlusion

**Fig. 1:**
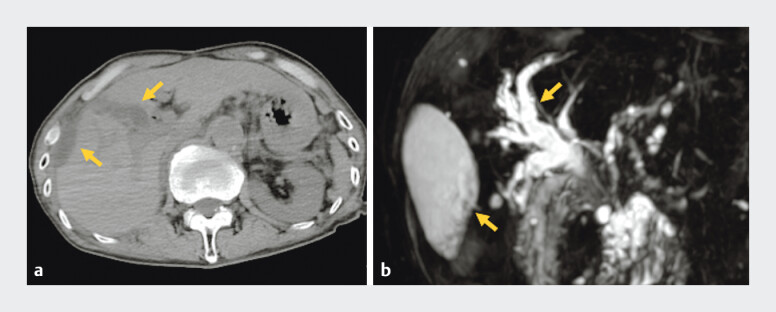
Computed tomography and magnetic resonance cholangiopancreatography showed a fluid collection on the liver surface and dilation of the right anterior hepatic duct (arrows).

**Fig. 2:**
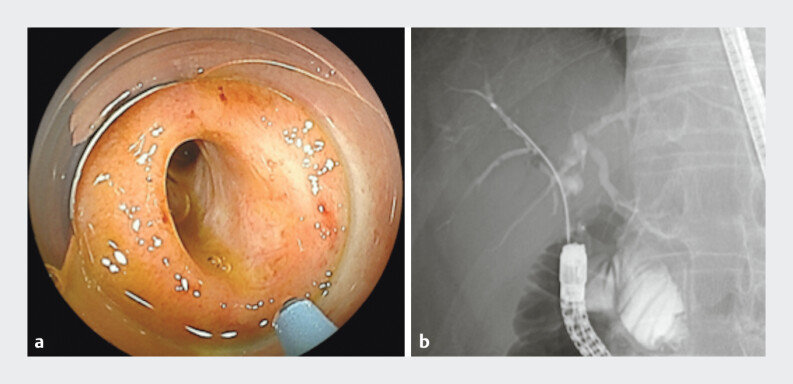
Although a double-balloon endoscope-assisted endoscopic retrograde cholangiopancreatography was performed, the right anterior hepatic duct could not be identified.

**Fig. 3:**
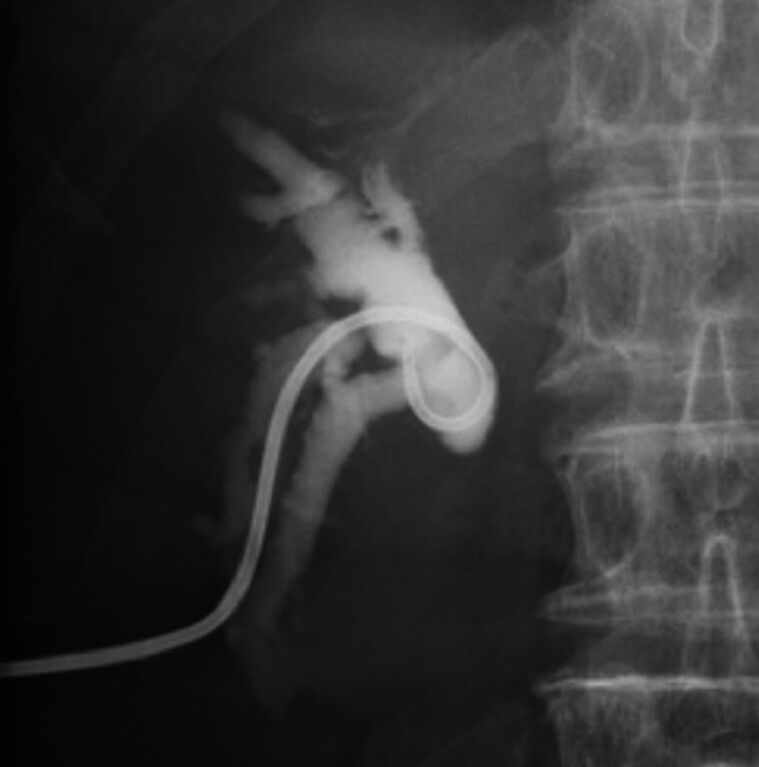
Percutaneous transhepatic biliary drainage was performed for the right anterior hepatic duct.

For the internal drainage, we attempted an endoscopic ultrasound (EUS)-guided hepaticojejunostomy. A forward-viewing echoendoscope (TGF-UC260J; Olympus, Tokyo, Japan) was inserted along a nasobiliary drainage tube placed in the left hepatic duct ( Video 1 ). After bile duct puncture with a fine-needle aspiration (FNA) needle, a guidewire was placed into B8 and the puncture site was dilated with a drill dilator (Tornus ES; Asahi Intec, Aichi, Japan). An additional guidewire was then placed into B5 using a double-lumen catheter (PIOLAX, Tokyo, Japan). The puncture site was dilated with a balloon catheter, followed by placement of a 7-Fr plastic stent and an 8-mm-diameter fully covered metal stent (M-Intraductal; Medico’s Hirata Inc., Osaka, Japan) ( Fig. 4 ). The PTBD catheter was removed and the patient was discharged two days postoperatively. Hepaticojejunostomy using a forward-viewing echoendoscope is a promising treatment option for a complete obstruction associated with a postoperative bile duct injury.

Endoscopic ultrasound-guided hepaticojejunostomy was performed using a forward-viewing echoendoscope. The dilated right anterior branch was punctured with a fine-needle aspiration needle, followed by dilation of the puncture site and stent placement.Video 1

**Fig. 4:**
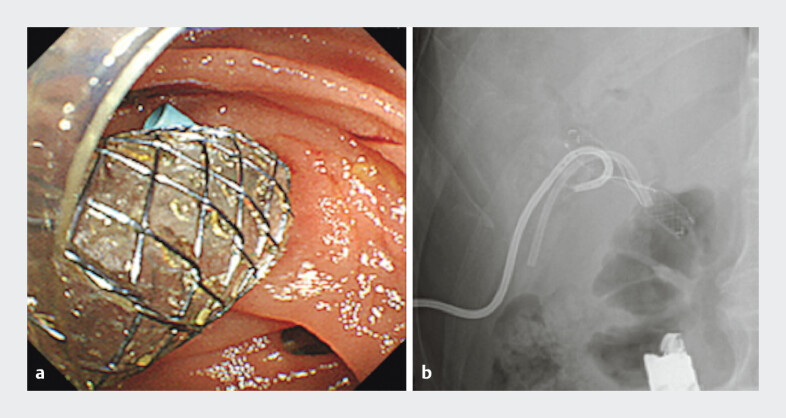
A 7-Fr plastic stent and an 8-mm-diameter fully covered metal stent were placed in a side-by-side fashion.

Endoscopy_UCTN_Code_TTT_1AS_2AH

## Notes

Endoscopy E-Videos https://eref.thieme.de/e-videos
*E-Videos* is an open access online section of the journal *Endoscopy* , reporting on interesting cases and new techniques in gastroenterological endoscopy. All papers include a high-quality video and are published with a Creative Commons CC-BY license. Endoscopy E-Videos qualify for HINARI discounts and waivers and eligibility is automatically checked during the submission process. We grant 100% waivers to articles whose corresponding authors are based in Group A countries and 50% waivers to those who are based in Group B countries as classified by Research4Life (see: https://www.research4life.org/access/eligibility/ ). This section has its own submission website at https://mc.manuscriptcentral.com/e-videos .
